# High Dielectric Tunability and Figure of Merit at Low Voltage in (001)-Oriented Epitaxial Tetragonal Pb_0.52_Zr_0.48_TiO_3_ Thin Films

**DOI:** 10.3390/nano15090695

**Published:** 2025-05-05

**Authors:** Hongwang Li, Chao Liu, Jun Ouyang

**Affiliations:** 1 Institute of Advanced Energy Materials and Chemistry, School of Chemistry and Chemical Engineering, Qilu University of Technology (Shandong Academy of Sciences), Jinan 250353, China; 10431220319@stu.qlu.edu.cn (H.L.); liuc@qlu.edu.cn (C.L.); 2 Key Laboratory of Key Film Materials & Application for Equipments (Hunan Province), Hunan Provincial Key Laboratory of Thin Film Materials and Devices, School of Material Sciences and Engineering, Xiangtan University, Xiangtan 411105, China

**Keywords:** Landau–Devonshire, thermodynamic, dielectric tunability, pulsed laser deposition (PLD), Pb(Zr_0.52_Ti_0.48_)O_3_(PZT)

## Abstract

Ferroelectric thin films with a high dielectric tunability (*η*) have great potential in electrically tunable applications, including microwave tunable devices such as phase shifters, filters, delay lines, etc. Using a modified Landau–Devonshire type thermodynamic potential, we show that the dielectric tunability *η* of a (001) tetragonal ferroelectric film can be analytically solved. After a survey of materials, a large *η* value above 60% was predicted to be achievable in a (001)-oriented tetragonal Pb(Zr_0.52_Ti_0.48_)O_3_ (PZT) film. Experimentally, (001)-oriented PZT thin films were prepared on LaNiO_3_-coated (100) SrTiO_3_ substrates by using pulsed laser deposition (PLD). These films exhibited good dielectric tunability (*η* ~ 67.6%) measured at a small electric field *E* of ~250 kV/cm (corresponding to 5 volts for a 200 nm thick film). It only dropped down to ~54.2% when *E* was further reduced to 125 kV/cm (2.5 volts for 200 nm film). The measured dielectric tunability *η* as functions of the applied electric field *E* and measuring frequency *f* are discussed for a 500 nm thick PZT film, with the former well described by the theoretical *η*(*E*) curves and the latter showing a weak frequency dependence. These observations validate our integrated approach rooted in a theoretical understanding.

## 1. Introduction

Dielectric materials with outstanding field tunable performance have become a focal point of research interest. The dielectric properties of these materials can be modulated by an external electric field, offering a broad range of potential applications, including phase shifters, tunable mixers, antennas, capacitors, and filters [1,2,3,4,5]. These applications provide significant support for the advancement of adaptable and reconfigurable electronic components. Tunable dielectrics are a type of functional materials whose dielectric permittivity (*χ*) can be adjusted by an external electric field (*E*). The pivotal parameter utilized to characterize tunable dielectrics is the so-called dielectric tunability *η*, which is defined as the relative change in the dielectric permittivity of the material under the influence of an electric field (*E*): *η* = [*χ*_0_ − *χ*(*E*)]/*χ*_0_, where *χ*_0_ is the zero field permittivity and *χ*(*E*) is the permittivity in the presence of an electric field *E*. Ferroelectric materials, as a typical nonlinear dielectric, often display high dielectric tunability, which has led to their broad utilization in devices that require adjustable dielectric properties. Due to their high and tunable dielectric permittivities, as well as mechanical and thermal stabilities, titanium-containing perovskite ferroelectric materials, such as (Ba,Sr)TiO_3_ [6,7,8], (Pb,Sr)TiO_3_ [9], (Pb,Ca)TiO_3_ [10], (Ba,Sn)TiO_3_, etc. [11], have attracted the majority of research interest in tunable dielectrics. These materials take different forms, including bulk ceramics [2,3,6] and thick [8] and thin films [4,5,7,9,10,11].

Due to the high operating voltages (often in thousands of volts) associated with their large thicknesses, bulk single-crystals or ceramics are not ideal for electrical tunable applications. Additionally, the high cost and the reliability issue from low dielectric breakdown strength (BDS) are also significant factors for their limited applications in tunable dielectrics. The high operating voltage is particularly problematic for integration of the bulk material into CMOS processed integrated circuits, which typically operate at voltages ~5 V [12] or below [13]. Fortunately, these issues can be resolved by employing thin film ferroelectric oxides, which offer a high dielectric tunability with an enhanced reliability under a much increased electric field. This is due to the thin films’ superior BDS, making them suitable for use in integrated tunable devices in a miniaturized scale.

Generally, the dielectric tunability *η* of a ferroelectric material increases with the magnitude of the applied electric field *E*. In the research of electrical tunable devices, the focus has been on achieving high tunability. Advanced thin film fabrication techniques, including rf sputtering [7,14], pulsed laser deposition [5,9,11,15], and chemical solution deposition [4,10], are used to prepare high-quality thin films with significantly enhanced BDS, allowing for higher tunabilities under high electric fields. For instance, back in 2003, a high *η* value of ~55% was achieved at an applied electric field *E* of 2400 kV/cm in Bi_1.5_Zn_1.0_Nb_1.5_O_7_ (BZN) thin films (*η* ~ 45% @ E = 1200 kV/cm), which were prepared using radio-frequency (RF) magnetron sputtering [14]. Furthermore, Pervez et al. reported ultra-high dielectric tunability *η* of ~84% under an electric field of 1 MV/cm in Ba_0.5_Sr_0.5_TiO_3_ films of ~120 nm thick, corresponding to an applied voltage of ~12 volts [7]. Recently (year of 2020), an exceptionally high *η* of around 85% was reported near room temperature under an ultra-high electric field of 2400 kV/cm (~24 volts) in the 0.5Ba(Ti_0.8_Zr_0.2_)O_3_-0.5(Ba_0.7_Ca_0.3_)TiO_3_ (BZT-BCT) epitaxial thin films (~100 nm thick). The films were deposited on a (110)-SrRuO_3_/SrTiO_3_ substrate using the pulsed laser deposition (PLD) technique [15]. One year later, Hao et al. showed a high room temperature *η* of ~77.4% under an electric field of 650 kV/cm in a 150 nm thick epitaxial K_0.5_Na_0.5_NbO_3_ (KNN) film sputtered on SrTiO_3_ substrates, corresponding to an applied voltage of 10 volts [16].

Although very large values of dielectric tunabilities (*η* > 60%) have been achieved in these research reports, a common shortcoming is that the desirable high *η* value requires a drive voltage significantly higher than the standard 5 V for CMOS circuitry, which limits the practical applications of these high-quality films. To address this issue, Hu et al. [17] introduced the concept of effective tunability *T*_0_ during their study of the electrical tunable Ba_0.6_Sr_0.4_O_3_ ceramics. $T_{0}=\frac{\eta }{E}$, where *η* is the dielectric tunability at a specific applied electric field *E* (*E* ≥ 0.5 kV/mm). This effective tunability can also be used as a reference parameter in the investigation of electrically tunable dielectric films. On the other hand, since dielectric loss tangent tan *δ* has a general trend of increasing with the dielectric tunability [18], the improvement in *η* is often accompanied by a boosted tan *δ* [19]. Therefore, researchers use another composite parameter called figure of merit (FOM) to evaluate the overall performance of an electrically tunable dielectric, which is given by FOM $=\frac{\eta }{\tan\;\delta }$ [17].

In our previous work, we used the modified Landau–Devonshire (LD)-type thermodynamic potential to derive a mathematical expression for the dielectric tunability of a (00l)-oriented tetragonal ferroelectric thin film [16]. We successfully designed strain-stabilized (00l) tetragonal KNN thin films with a high dielectric tunability. The measured maximum dielectric tunability (*η*) (75~80%) agreed well with the theoretically calculated values. Moreover, the theoretically predicted tunability as a function of electric field *η*(*E*) agreed fairly well with the measured *η*-*E* curves. These results confirm that the dielectric tunability of a ferroelectric thin film can be predicted through computational methods [20]. However, a relatively large operating voltage/electric field (10 V or 650 kV/cm), as well as a sizable loss tangent (~0.1), prevents these films from practical applications.

In this work, we use a similar theoretical approach (LD thermodynamic potential) to predict the dielectric tunability performance of a tetragonal PZT (52/48) thin film, which turns out to possess both large tunability *η* and high effective tunability $T_{0}$. This work combines a modified Landau–Devonshire model with pulsed laser deposition to design and validate high-performance PZT films for low-voltage tunable dielectric applications. Experimentally, we have successfully fabricated high-quality epitaxial tetragonal PZT (52/48) films on LaNiO_3_-coated SrTiO_3_ substrates with a (00l)-orientation, using a pulsed laser deposition process. The dielectric tunability factor ($\lambda_{f}$) of these films is ~2.5 × 10^−7^ mV^−1^, and under an external electric field of 200 kV/cm (10 V on a 500 nm thick film) or 250 kV/cm (5 V on a 200 nm thick film), the corresponding dielectric tunability is ~58.8% (@200 kV/cm) and ~67.6% (@250 kV/cm), respectively. The dielectric loss tangents at zero field are relatively low (~0.012 @ 1 kHz and 0.047 @ 1 MHz for the 500 nm film), which have led to significantly improved FOM for PZT films. Compared to (111)- or (110)-oriented PZT films, (00l)-oriented epitaxial films exhibit enhanced polarization alignment along the out-of-plane direction. Therefore, (00l)-oriented PZT films have demonstrated superior tunability due to their reduced domain wall pinning and higher remnant polarization compared to randomly oriented or polycrystalline counterparts [16,21]. These results are quite significant for the films’ applications in electrically tunable and highly integrated micro-devices.

## 2. Materials and Methods

### 2.1. Dielectric Tunability of a (00l)-Oriented Tetragonal Ferroelectric Film

According to the Landau–Devonshire (LD) thermodynamic theory [22], the relationship between the dielectric permittivity $\chi^{f}(E)$ (in the presence of an electric field *E*) of a (00l)-oriented epitaxial tetragonal ferroelectric film and the electric field *E* can be expressed as follows [16]:

(1) $$\chi^{f}\left(E\right)=\frac{dp}{dE}\approx \frac{\chi_{0}^{f}}{\sqrt{1+\lambda_{f}E}}$$

This formula reveals the nonlinear nature of the dielectric permittivity of a ferroelectric film. Here, $\lambda_{f}$ is the dielectric tuning factor of the film, representing the rate of nonlinear change in the film’s dielectric permittivity, and $\chi_{0}^{f}$ is the film’s zero field permittivity. The expression for $\lambda_{f}$ is given by the following [16]:

(2) $$\lambda_{f}=4{\left(\chi_{0}^{f}\right)}^{2}•[3P_{0}^{f}\beta +10(P_{0}^{f}{)}^{3}\alpha_{3}]$$

where $P_{0}^{f}$ is the film’s remnant polarization, while *β* and $\alpha_{3}$ are material dependent parameters consisting of LD thermodynamic potential coefficients (“Landau coefficients”) and electromechanical coefficients (elastic/electrostrictive) (see Table 1). The dielectric tunability of the film, *η*, can then be represented as

(3) $$\eta =\frac{\chi_{0}^{f}-\chi^{f}(E)}{\chi_{0}^{f}}=1-\frac{1}{\sqrt{1+\lambda_{f}E}}$$

The LD model is applicable under conditions where the electric field does not induce irreversible domain reconfigurations or dielectric breakdown. For the PZT films studied here, the model remains valid since we were fitting the dielectric permittivity–electric field curve $\chi^{f}(E)$ from the high field end to zero field (i.e., in the well poled, equilibrium state).

From Equation (3), it can be seen that the dielectric tunability *η* is positively correlated with the dielectric tuning factor $\lambda_{f}$. Under a given external electric field, the larger the $\lambda_{f}$, the greater the tunability *η*. When the material parameters *α*_11_, *α*_111_, *S*, and *Q*_12_ are all known, both $\lambda_{f}$ and *η* can be determined from the zero-field dielectric permittivity $\chi_{0}^{f}$ and polarization $P_{0}^{f}$ ($\chi_{0}^{f}$ and $P_{0}^{f}$ are obtained from experimental measurements). Theoretically, $\lambda_{f}$, $\chi_{0}^{f}$, and $P_{0}^{f}$ are all functions of the misfit strain $\varepsilon_{M}^{0}$. When the material’s Landau coefficients *α*_1_, *α*_11_, *α*_111_; electrical polarization/elastic coefficients/electrostrictive coefficient −$P_{0}^{b}$, *S*_11_/*S*_12_, and *Q*_12_; and the misfit strain $\varepsilon_{M}^{0}$ are all known, $\lambda_{f}$, $\chi_{0}^{f}$, and $P_{0}^{f}$ can all be accurately calculated. Here, $\varepsilon_{M}^{0}$ represents the in-plane misfit strain of the film, which is the difference between the lattice constant of the substrate-clamped film and that of the free-standing film (similar to that of the bulk ceramic or single crystal). *Q*_12_ denotes the electrostrictive coefficient, while $P_{0}^{b}$ and $P_{0}^{f}$ represent the remnant polarization of the bulk and thin film, respectively. *α*_1_, *α*_11_, and *α*_111_ represent the first-, second-, and third-order Landau coefficients of the thin film material under stress-free conditions. S denotes the in-plane elastic modulus of the thin film. *S* = $S_{11}^{p}$ + $S_{12}^{p}$, with ${\;S}_{11}^{p}$ and $S_{12}^{p}$ being the elastic coefficients of the cubic-phase bulk material. These material parameters can be obtained through experimental measurements [24].

Theoretical analysis reveals that strain-free films exhibit lower dielectric tunability due to weaker polarization nonlinearity. Strain engineering, achieved via substrate lattice mismatch, enhances tunability by stabilizing tetragonal phases and increasing polarization anisotropy. Specifically, tensile strain (as implemented in our films) enhances in-plane polarization alignment, thereby amplifying dielectric permittivity and its tunability. In contrast, compressive strain promotes out-of-plane polarization alignment, leading to a reduction in dielectric permittivity and tunability. These distinct strain-dependent trends align with prior studies on strain-mediated ferroelectricity [16,25].

### 2.2. Deposition of the PZT Film

The SrTiO_3_ (STO) substrates (10 mm × 10 mm) and ceramic targets of Pb(Zr_0.52_Ti_0.48_)O_3_ (25 mm × 5 mm) and LaNiO_3_ (*Φ* = 50 mm*, t* = 3 cm) were purchased from Anhui Institute of Optics, Chinese Academy of Science. To prepare the epitaxial PZT films, LaNiO_3_ films with a thickness of ~150 nm were pre-deposited on (00l)-oriented STO single crystalline substrates. LaNiO_3_ serves as a conductive bottom electrode with perovskite structure, ensuring epitaxial growth of PZT on SrTiO_3_. Its lattice parameters (~3.84 Å) bridge the mismatch between STO (3.905 Å) and PZT (~4.04 Å), minimizing interfacial defects [26]. Such a deposition was carried out via an RF magnetron sputtering process at 500 °C in a mixed Ar/O_2_ atmosphere (0.3 Pa, Ar/O_2_ flow ratio = 4:1). Then, 200 nm and 500 nm thick PZT films were grown on the LaNiO_3_/SrTiO_3_ substrates via pulsed laser deposition using a KrF excimer laser (248 nm wavelength). This technique is well known to be capable of producing high-quality epitaxial thin films [5,9,11,15]. For the PZT film in the current study, the optimum deposition conditions include a substrate temperature of 600 °C, an oxygen pressure of 10 Pa, a 500 mJ/pulse fluence of the laser beam, a laser frequency of 10 Hz, and a substrate–target distance of 60 mm. After deposition of the PZT film, the substrate temperature was decreased to room temperature at a rate of 20 °C/min. The cooling down of the thin film heterostructure was carried out in an oxygen atmosphere of 1 × 10^4^ Pa in order to suppress the generation of oxygen vacancies. After the PZT film had been taken out from the vacuum chamber, dot-shaped Au top electrodes (*Φ* = 0.2 mm) were sputtered at room temperature via a shadow mask on the surface of the PZT films.

### 2.3. Characterization of the PZT Films

X-ray diffraction (XRD) was used to analyze the crystalline quality of the PZT films in a SmartLab^TM^ 9 kW X-ray diffractometer (Rigaku, Tokyo, Japan). Atomic force microscopy (AFM) and piezoelectric force microscopy (PFM) were used to analyze the surface morphology and reveal the ferroelectric nature of the PZT films using an AFM100 Plus microscope (Hitachi, Tokyo, Japan). The thicknesses and nanostructures of the Pb(Zr_0.52_Ti_0.48_)O_3_ thin film heterostructures were analyzed via transmission electron microscopy (TEM), using a Talos F200X G2 microscope (Thermo Fisher Scientific, Waltham, MA, USA). The polarization–electric field (*P*-*E*) hysteresis loops and the leakage current density (*J*-*E*) curves were measured by using a Multiferroic II ferroelectric tester (Radiant Technology, El Segundo, CA, USA). The frequency- and dc bias-dependent dielectric properties (*C*-*f*, *C*-*V*) of the PZT thin films were measured using a TH2828H LCR bridge (Tonghui Electronics, ChangZhou, China). The *C*-*f* tests were conducted in the frequency range of [1 kHz, 1 MHz], while the *C*-*V* tests were carried out by superimposing a small AC signal (*V*_p-p_ = 0.5 V) on a DC bias voltage sweeping from its negative maximum to its positive maximum, and vice versa.

## 3. Results and Discussion

**Figure 1:**
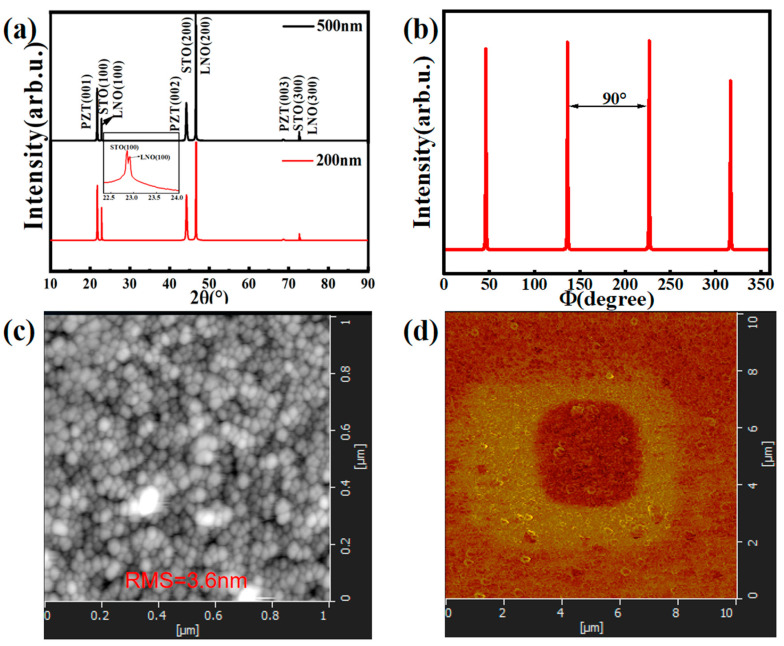
Microstructure and surface morphology of the PZT films. (**a**). X-ray diffraction (XRD) 2*θ*-scan pattern of the 500 nm and 200 nm thick Pb(Zr_0.52_Ti_0.48_)O_3_ thin films; (**b**). XRD *Φ* scans of the (101) reflections of the 500 nm film in (**a**); (**c**,**d**). AFM and PFM images of the 500 nm PZT film.

**Figure 2:**
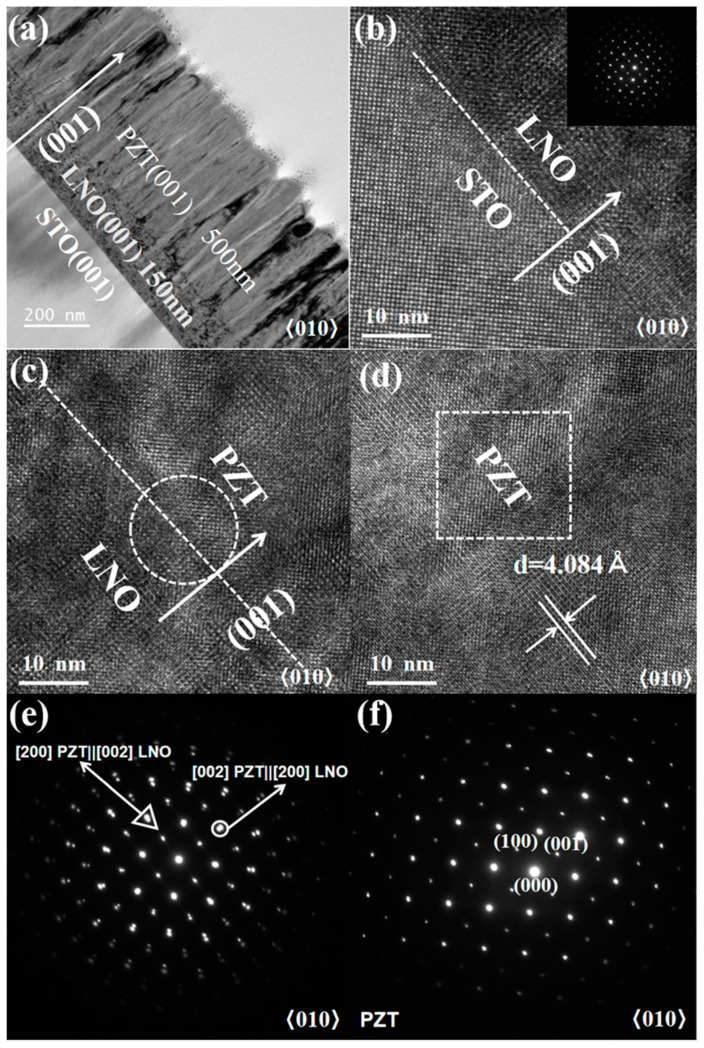
Cross-sectional TEM analyses of the 500 nm thick PZT film. (**a**) Cross-sectional bright-field TEM image of the PZT/LNO/STO heterostructure; (**b**,**c**) are the high-resolution TEM images taken near the LNO/STO and PZT/LNO interfaces; (**e**) and (**f**) are the selected area electron diffraction patterns (SAEDs) taken from (**c**) and (**d**), respectively (areas schematically shown by the boxes).

Figure 1a shows the XRD 2*θ*-scan patterns for the 500 nm and 200 nm thick PZT films. Only sharp {00l} diffraction peaks are observed for the PZT films, together with the neighboring {l00} ones from the STO substrate, indicating a pure perovskite phase with a (00l) orientation. It is noted that the PZT film has a tetragonal symmetry promoted by the LNO-coated STO substrate. LNO peaks are very close to those of STO substrate. The XRD pattern near the (100) LNO peak is shown as the inset of Figure 1a to reveal its existence and indication of good crystallinity (also confirmed by the TEM analysis in Figure 2). A 4.084 Å out-of-plane lattice parameter was measured for the 500 nm film, while it was ~4.077 Å for the 200 nm film. These lattice parameters correspond to in-plane tensile strains of ~1.35% (500 nm film) and ~1.52% (200 nm film), respectively. ($\varepsilon_{xx}$ = $\varepsilon_{yy}$ = $-\frac{1-\nu }{2\nu }\varepsilon_{zz}$, $\varepsilon_{zz}$ is the out-of-plane strain, v is the Poisson’s ratio, which is ~$\frac{1}{3}$ for PZT. $\varepsilon_{zz}$ = $\frac{C_{\mathrm{film}}-C_{\mathrm{bulk}}}{C_{\mathrm{bulk}}}$, where $C_{\mathrm{film}}$ and $C_{\mathrm{bulk}}$ are the out-of-plane lattice parameters of the PZT film and bulk ceramic. The reference $C_{\mathrm{bulk}}$ data for tetragonal Pb(Zr_0.52_Ti_0.48_)O_3_ is 4.14 Å [27]. Moreover, the *Φ*-scan pattern of the (101) reflection of the 500 nm PZT film is shown in Figure 1b. Four strong reflection peaks are equally separated by 90°, indicating a fourfold symmetry of the film normal and an epitaxial quality of the PZT film. The 200 nm thick PZT film showed a similar *Φ*-scan pattern of its (101) reflection. Meanwhile, Figure 1c, d display the AFM surface scan and out-of-plane PFM poling–reverse poling (with +5 V/−5 V voltages) images of the 500 nm PZT film. Figure 1c shows a smooth film surface with a root mean square (RMS) surface roughness of ~3.6 nm, while Figure 1d clearly reveals the ferroelectric nature of the PZT film with its polarization aligned out of plane, a (00l)-oriented film normal.

Figure 2a is a representative cross-sectional bright-field TEM image of the 500 nm PZT film, which reveal clean and sharp interfaces in the PZT/LNO/STO heterostructure, as well as a homogeneous and dense film morphology. In the high-resolution TEM images depicted in Figure 2b,c, the interfaces between the STO (100) substrate and the LNO layer, and between the LNO layer and the PZT film (marked with dashed lines) are shown, indicating excellent lattice matching between these materials. As shown in the inset of Figure 2b, the selected area electron diffraction pattern (SAED) taken at the STO/LNO interface showed coherent growth of the (100) LNO layer on the (100) STO substrate. Furthermore, a cube-on-cube epitaxial growth of the (00l) perovskite PZT film on LNO was evidenced by the SAED shown in Figure 2e, which was taken at the LNO/PZT interface. The in-plane and out-of-plane orientation relationships in the PZT/LNO/STO thin film heterostructure can be described as [010]PZT||[010]LNO|| [010]STO, [100]PZT||[001]LNO||[001]STO, and [001]PZT||[100]LNO||[100]STO. Lastly, in Figure 2d, the PZT film shows a single-crystalline quality with directly visible (00l) lattice planes, whose plane spacing was measured to be ~4.084 Å, consistent with the XRD result. Figure 2f is the SAED taken from inside the PZT film, which shows a single set of sharp diffraction spots for PZT, as compared with the mixed sets of diffraction spots (LNO and PZT) shown in Figure 2e. In Figure 2f, the in-plane (100) and out-of-plane (00l) diffraction spots are clearly distinguishable, with the in-plane (100) spacing from the (000) central spot being longer in the reciprocal space.

**Figure 3:**
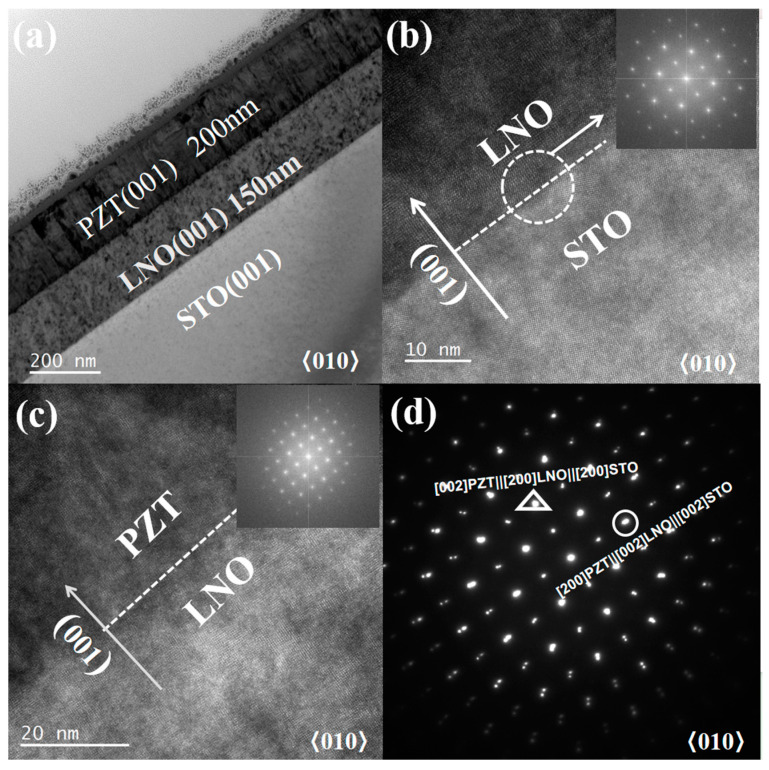
Cross-sectional TEM analyses of the 200 nm thick PZT film. (**a**) Cross-sectional bright-field TEM image of the PZT/LNO/STO heterostructure; (**b**,**c**) are the high-resolution TEM images taken near the LNO/STO and PZT/LNO interfaces; (**d**) is the electron diffraction pattern of the whole heterostructure, including diffraction spots from PZT, LNO, and STO.

For the 200 nm PZT film, Figure 3a presents a homogeneous, dense thin film heterostructure with clean and sharp interfaces at both the PZT/LNO and LNO/STO boundaries, maintaining the high-quality epitaxial growth mode observed in the 500 nm film. Despite the reduced thickness, the PZT layer exhibits a dense film morphology. High-resolution TEM images (Figure 3b,c) reveal excellent lattice matching at the STO/LNO and LNO/PZT interfaces, with no evidence of interfacial dislocations or grain boundaries. The FFT-SAED pattern (inset of Figure 3b) confirms a coherent growth of (100) LNO on STO (100), preserving the same crystallographic registry as in Figure 2b. An FFT-SAED pattern (inset of Figure 3c) taken at the LNO/PZT interface demonstrates an epitaxial alignment of the PZT (00l) plane on (100) LNO. Overall, the epitaxial relationships remain the same as in the case of the 500 nm film: in-plane:[010]PZT‖[010]LNO‖[010]STO and [100]PZT‖[001]LNO‖[001]STO; out-of-plane: [001]PZT‖[100]LNO‖[100]STO.

**Figure 4:**
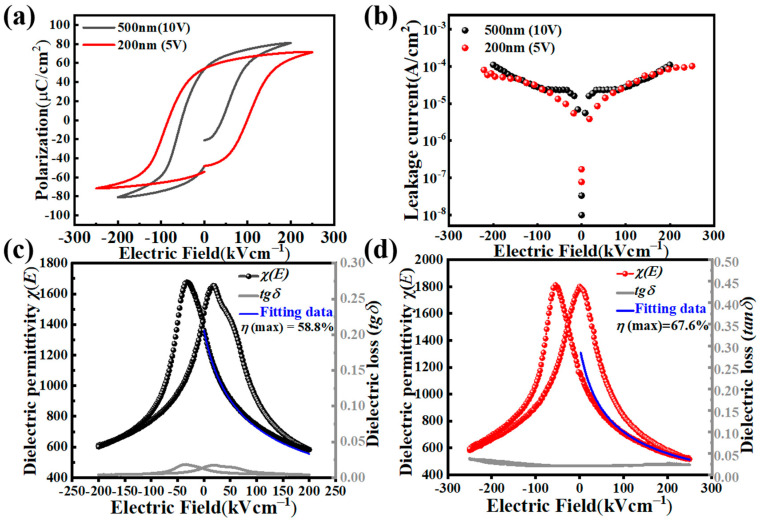
Electrical performance of the PZT films: (**a**) *P*-*E* hysteresis loops; (**b**) leakage current density (*J*-*E*) curves; (**c**,**d**) measured and computed dielectric permittivity *χ*-electric field *E* (*χ*-*E*) curves of (**c**) the 500 nm PZT film and (**d**) the 200 nm PZT film. The dielectric loss tangent (tan *δ*)–electric field *E* (tan *δ*−*E*) curves were also shown.

**Figure 5:**
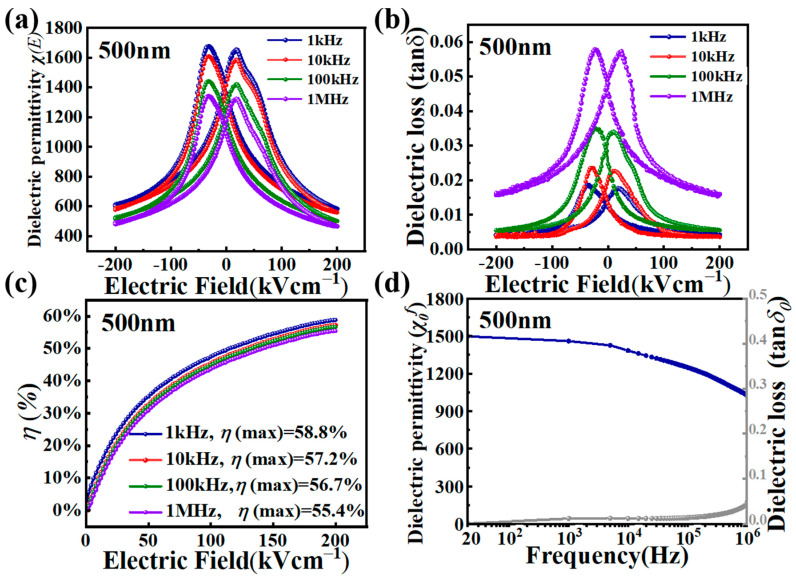
Dielectric properties of the 500 nm PZT films as a function of measuring frequency. (**a**) Dielectric permittivity *χ*(*E*). (**b**) Dielectric loss tangent tan *δ*(*E*). (**c**) Dielectric tunability *η*(*E*) measured at different frequencies (1 kHz to 1 MHz) as a function of the dc bias field *E*. (**d**) Dielectric permittivity *χ*_0_*^f^* and loss tangent tan *δ* (@ zero dc bias) as a function of the measuring frequency.

Figure 4a shows the *P*-*E* hysteresis loops of the two PZT films measured @1 kHz. While the remnant polarization *P*_r_ in the 500 nm film is approximately 53.5 μC/cm^2^, that of the 200 nm PZT film is slightly lower (~50 μC/cm^2^). Polarization hysteresis loops were measured within a ±5% relative error (via repeated sweeps). The 200 nm film’s remnant polarization *P*_r_ ~ 50 μC/cm^2^ was about 6.5% lower than that of the 500 nm film (53.5 μC/cm^2^); therefore, its higher tensile strain (1.52% vs. 1.35%) did play a part in its lower *P*_r_ (tensile strain suppressing out-of-plane polarization) [28,29]. The 200 nm film requires half of the voltage (5 V) as that for the 500 nm film (10 V) to achieve polarization saturation (and hence a large tunability); therefore, it is more suitable for low-voltage tunable dielectric applications. The large remnant polarization of the PZT films and their easy saturation with an applied electric field have formed the basis for their electrical tunable applications. Furthermore, while the coercive field (~100 kV/cm) of the 200 nm film is higher than that of bulk PZT, its operating voltage (5 V, or 2.5 Vc) remains compatible with CMOS operating requirement. Figure 4b displays the leakage current density (*J*-*E*) curves of the two PZT films. From the figure, it can be seen that at an electric field of ~100 kV/cm (5 V for the 500 nm film, 2 V for the 200 nm film), the leakage current density is in the low 10^−5^ A/cm^2^. Only under the maximum applied electric field of 200–250 kV/cm (10 V for the 500 nm film, 5 V for the 200 nm film), the leakage current density reached the level of 10^−4^ A/cm^−2^. These results indicate that PZT films are good insulators, a prerequisite for their applications in tunable dielectric applications. Consequently, the room temperature dielectric properties (dielectric permittivity *χ* and loss tangent tan *δ*) as functions of a direct current (dc) bias field were measured, which were conducted by superimposing an AC signal with a small amplitude (*V*_p-p_ = 0.5 V in this work) on a sweeping DC bias field. The DC bias was swept from its maximum positive value to the maximum negative one and vice versa. Figure 4c,d are the *χ*(*E*) and tan *δ*(*E*) curves of the 500 nm and 200 nm PZT films, respectively, both measured at the same ac frequency of 1 kHz. For the 500 nm film, its zero-field dielectric permittivity *χ*_0_*^f^* is ~1416, while under the maximum dc bias of 200 kV/cm, the dielectric permittivity *χ*(*E*) dropped to ~583, corresponding to a dielectric tunability *η*(max) ~58.8%. With a small dielectric loss of ~0.012 at zero field, the FOM for the 500 nm PZT film as an electrically tunable dielectric is 49. On the other hand, for the 200 nm film, the apparent *χ*_0_*^f^* is ~1160 for its positively poled state, while under the maximum dc bias of 250 kV/cm, the dielectric permittivity *χ*(*E*) dropped to ~516, corresponding to a dielectric tunability *η*(+max) ~55.5%. However, the 200 nm film had a built-in field *E*_bi_ of ~+27.5 kV/cm, as shown from its *P*-*E* loop in Figure 4a. On its negatively poled state, the *χ*_0_*^f^* is ~1802 and *χ*(*E*) ~583 at *E* = −250 kV/cm, leading to a higher dielectric tunability *η*(-max) ~67.6%. Meanwhile, the dielectric loss for the thin PZT film is ~0.021 at zero field, resulting in a decent FOM value (~26.43 or ~32). Moreover, when evaluated with the effective tunability $T_{0}$, both PZT films showed excellent performance: $T_{0}$ = 0.0294 (kV/mm)^−1^ for the 500 nm film, and 0.0207/0.00269 (kV/cm)^−1^ for the 200 nm film. Lastly, the theoretically predicted *χ*(*E*) curves for the two PZT films were plotted (in blue) together with the experimental curves in Figure 4c,d, using Equations (1) and (2). These *χ*(*E*) curves fit fairly well with the experimental data. While low-frequency measurements (1 kHz) can include interfacial artifacts, such as contact effects, interface polarization effects, etc., inflating the measured dielectric permittivity [30], our high-frequency dielectric data (up to 1 MHz), as seen in Figure 5, displayed a consistent tunability trend, confirming a dominant intrinsic PZT behavior. This weak frequency dependence of the dielectric permittivity, together with the low leakage currents (Figure 4b, <10^−4^ A/cm^2^ @200 kV/cm), have confirmed a suppressed parasitic contribution. These results are consistent with prior reports on epitaxial films [9,15,26].

Frequency-dependent dielectric properties, including dielectric permittivity *χ*, loss tangent tan *δ*, and dielectric tunability *η*, were investigated for the 500 nm PZT film, given its low dielectric loss performance. Figure 5a,b display the *χ*(*E*) and tan *δ*(*E*) curves sweeping from +200 kV/cm to −200 kV/cm and vice versa, for the measuring frequencies of 1 kHz, 10 kHz, 100 kHz, and 1 MHz. As the frequency was increased from 1 kHz, the zero-field dielectric permittivity *χ*_0_*^f^* decreased from 1416 kHz (@1 kHz) to 1360 (@10 kHz), 1165 (@100 kHz), and finally to 1088 (@ 1 MHz) [31]. Meanwhile, the field-tunable dielectric permittivity *χ*(*E*) also decreased with an increasing measuring frequency. At the maximum electric field *E* = 200 kV, *χ*(*E*) shows its smallest value from 583 @ 1 kHz to 575 @10 kHz, 502 @100 kHz, and finally to 485 @1 MHz. Consequently, the dielectric tunability *η* only showed a small change with the measuring frequency leaping across three orders of magnitude. Under *E* = 200 kV/cm, *η* is 58.8% under 1 kHz, 57.2% under 10 kHz, 56.7% under 100 kHz, and finally 55.4% under 1 MHz. Such a weak frequency dependence of *η* is clearly displayed in Figure 5c, where *η* as functions of the applied electric field *E* were plotted for the four measuring frequencies. The four *η*(*E*) curves are close to each other in the full range of *E*, i.e., [0, 200 kV/cm]. On the other hand, the dielectric loss tangent tan *δ* showed a significant change with increasing measuring frequency. As shown in Figure 5b, the dielectric loss tangents from the three low frequency measurements are close, especially at the high field end. While the zero field tan *δ* showed a slight increase from 0.012 (@1 kHz) to 0.019 (@10 kHz) and 0.027 (@100 kHz), those at *E* = 200 kV/cm showed similar values at 0.0036 (@1 kHz), 0.0042 (@10 kHz), and 0.0055 (@100 kHz), respectively. This weak frequency dependence can be attributed to the high-quality epitaxial hetero-interfaces in the PZT film, which have led to reduced interfacial charged defects and hence a suppressed interfacial polarization relaxation taking place in such a frequency range [32,33]. On the other hand, dielectric loss measured at 1 MHz showed much higher tan *δ* values, reaching 0.047 at zero field and 0.016 at the high field end of *E* = 200 kV/cm. The rise in tan *δ* near 1 MHz indicates bulk charge carrier relaxations, which can include those from oxygen vacancies, which lead to significant bulk polarization relaxation [32,33] and hence a boosted dielectric loss. However, overall low tan *δ* (<0.05) values across the whole frequency range (1 kHz to 1 MHz) confirm a minimal defect impact on tunability, which is supported by PLD’s oxygen-rich growth conditions and consistent with the results shown in Figure 5a–c. Lastly, in Figure 5d, the zero-field dielectric permittivity *χ*_0_*^f^* and loss tangent tan *δ*_0_ of the 500 nm PZT film were plotted as functions of the measuring frequency *f*. The test frequency range was from 20 Hz to 1 MHz. The dielectric permittivity decreased from 1498 to 1028 with the frequency increasing from 20 Hz to 1 MHz. The dielectric loss tangent tan *δ*_0_ was about 0.0016–0.044, which increased with frequency in the range from 20 Hz to 1 MHz. Below 100 kHz, tan *δ* remained low and only slightly increased with frequency. This indicates that the film’s polarization was able to follow the changes in the alternating electric field at low frequencies, i.e., a suppressed (interfacial-dominated) polarization relaxation, as revealed by Figure 5b.

**Figure 6:**
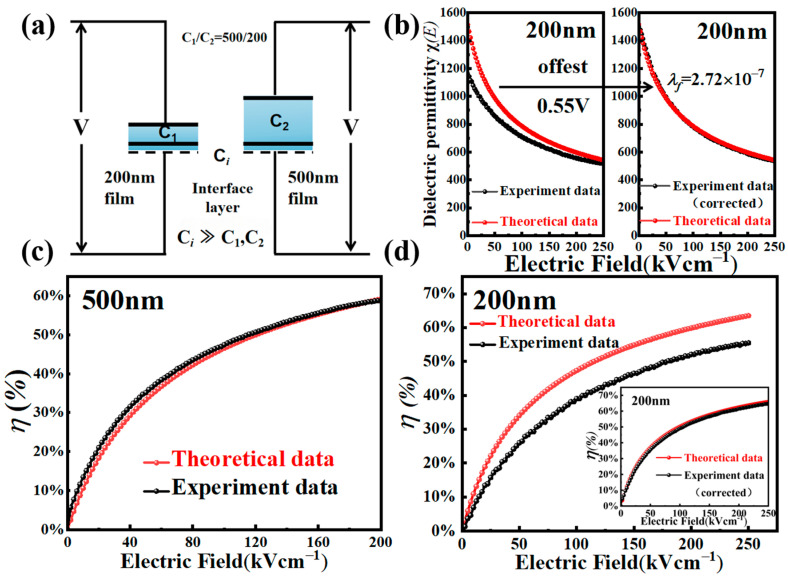
Modeling of the dielectric tunability of the PZT films (**a**) the “interface layer” model. (**b**) Fitting of a small offset voltage in the *χ*(*E*) curve of the 200 nm thick PZT film to compensate for the voltage drop across its interface layer. (**c**,**d**) Dielectric tunability *η*(*E*) curves computed and measured at 1 kHz for (**c**) the 500 nm and (**d**) the 200 nm thick PZT films (with the “interface layer” correction), respectively.

Figure 6a presents a simple model of serially connected capacitors [16] to illustrate the impact of film thickness on the interfacial layer voltage drop, which accounts for the built-in field shown in the *P*-*E* and *χ*-*E* curves of the 200 nm film. The interfacial “dead layer” is about the same in both PZT films. It is very thin [34] and has a large capacitance over that of the PZT film. When the PZT film is sufficiently thick, its capacitance becomes negligibly small compared to that of the interfacial layer, resulting in a nearly complete application of the external voltage across the PZT film. However, when the film thickness was reduced to 200 nm, the capacitance of the PZT film increased substantially, and the voltage drop across the “interfacial capacitor” became non-negligible. Consequently, this interfacial voltage drop induced a built-in field in the 200 nm PZT film. A fitting to the theoretical *χ*(*E*) by offsetting the experimental *χ*-*E* curve showed an interfacial voltage drop of 0.55 V under an applied voltage of 5 V (*E* = 250 kV/cm, Figure 6b), which is consistent with the +27.5 kV/cm built-in field shown by Figure 4a,d. The interfacial voltage drop (*V*_int_ = 0.55 V) was determined by fitting the experimental *χ*(*E*) curve to the theoretical model using a serially connected capacitor model. The voltage ratio *V*_int_/*V*_app_ = *C*_PZT_/(*C*_int_ + *C*_PZT_) only becomes non-negligible when the PZT film thickness is small (Figure 6a). Fitting from the built-in field of the *P*-*E* curve yielded a 0.55 V voltage drop across the interface layer for the 200 nm film [34]. Lastly, Figure 6c,d show the experimentally measured and theoretically predicted dielectric tunability versus electric field (*η*-*E*) curves for the 500 nm and 200 nm PZT films. The experimental *η*(*E*) were derived using the original data sets of dielectric permittivity *χ*(*E*), with an “interface layer” correction being applied to that of the 200 nm PZT film (Figure 6d). On the other hand, the theoretical *η*(*E*) curves were computed by using Equation (3) and Table 1. The experimental and theoretical *η*(*E*) curves agree well for the two PZT films, particularly at the high electric field end [34]. It is noted that these dielectric tunability curves exhibit a saturation behavior with an increasing electric field, indicating a possible trade-off between tunability and working voltage/field.

As shown in Table 2, the dielectric tunability performance (*η*, *T*_0_, FOM) of our PZT films under a low driving voltage is significantly better than those of the electrically tunable dielectric films reported previously [11,14,30,35,36,37,38,39]. Under the applied voltage of 10 V, the 500 nm PZT film showed an outstanding dielectric tunability performance (*η* ~ 55.4% to 58.8%, *T*_0_ ~ 0.0277 to 0.0294, FOM = 49 @ 1 kHz and =11.79 @ 1 MHz). When the voltage was reduced to 5 V, it still showed excellent dielectric tunability performance (*η* ~ 43.5% to 47.0%, *T*_0_ ~ 0.0435 to 0.047, FOM = 39.2 @ 1 kHz and =9.06 @ 1 MHz). It should be noted that these dielectric tunability performance is only weakly frequency dependent in the measuring frequency range of [1 kHz, 1 MHz]. Furthermore, under a very low voltage level of 2.5 volts, the 200 nm PZT film showed an outstanding dielectric tunability performance (*η* ~ 53.9%, *T*_0_ ~ 0.0431, FOM = 25.67, all @ 1 kHz). Future work is under way to improve the PZT thin film’s quality to allow for it work properly under higher frequencies.

## 4. Conclusions

In this work, (00l)-oriented tetragonal PZT thin films with high dielectric tunability were successfully designed via a computational approach using an LD-type thermodynamic potential. PZT thin films with thicknesses of 500 nm and 200 nm were deposited onto LaNiO_3_-buffered (00l) SrTiO_3_ substrate via pulsed laser deposition. The typical small signal dielectric constant and loss tangent at a frequency of 1 kHz were 1400 and 0.012, respectively. At room temperature and 200 kV/cm (10 volts on the 500 nm film), the tunability *η*, normalized tunability *T*_0_, and figure of merit are 58.8%, 0.0294 (kV/mm)^−1^, and 49, respectively. The measured maximum dielectric tunability *η* (58.8%) is in good agreement with the computed theoretical value. Furthermore, when we reduced the operating voltage down to 5 V, the 500 nm PZT film still showed an excellent dielectric tunability performance (*η*, *T*_0_, FOM) across a broad frequency range of [1 kHz, 1 MHz]. Lastly, in the thin PZT film (200 nm thick), we demonstrated the possibility of achieving good tunable performance under very low voltage (2.5 V). These results suggest that PZT thin films are promising material candidates in electrically tunable devices.

## Figures and Tables

**Table 1:** Parameters of PZT films used in theoretical computations of this work [16,23].

Parameters	*α*_1_ (10^7^)	*α*_11_ (10^8^)	*α*_111_ (10^9^)	*Q*_12_	$P_{0}^{b}$	$P_{0}^{f}$	$\varepsilon_{M}^{0}$		*S* $=S_{11}^{p}$ $+S_{12}^{p}$
	−4.887	0.4764	1.336	−0.046	0.5	0.535	0.0135		8.3 × 10^−12^
$\alpha (10^{7})=\left\{\alpha_{1}-\frac{2[\varepsilon_{M}^{0}+Q_{12}{\left(P_{0}^{b}\right)}^{2}]Q_{12}}{S}\right\}$		$\beta (10^{9})=4\left(\alpha_{11}+\frac{Q_{12}^{2}}{S}\right)$		$\alpha_{3}(10^{9})=6(\alpha_{111})$		$\lambda_{f}\left(10^{-7}\right)=4{\left(\chi_{0}^{f}\right)}^{2}•[3P_{0}^{f}\beta +10(P_{0}^{f}{)}^{3}\alpha_{3}]$			
Units		C^−2^m^2^ N		C^−4^m^6^ N		C^−6^m^10^ N		mV^−1^	
		−5.374		1.205		8.016		2.5	

**Table 2:** Dielectric tunability performances of typical ferroelectric films.

Sample	Orientation	*E*-Field (kV/mm)/ Frequency	*η;* Tunability (%) @ Voltage (Volts)	*T*_0_ (mm/kV)	FOM	Ref. No
PZT (52/48) 500 nm thick (this work)	001	20 @1 kHz 10@1 kHz	58.8% @10 V 47.0% @5 V	0.0294 0.047	49 39.2	-
		20 @1 MHz 10 @1 MHz	55.4% @ 10 V 43.5% @5 V	0.0277 0.0435	11.79 9.06	-
PZT (52/48) 200 nm thick (this work)	001	25 @1 kHz 12.5 @1 kHz	67.6% @ 5 V 54.2% @2.5 V Note: *η*(−max) data	0.0269 0.0431	32 25.67	-
BSSnT 300 nm thick	100	30 @10 kHz 15 @10 kHz	31% @8 V 13.2% @4 V	0.0103 0.0088	6.2 2.64	[11]
Bi_1.5_Zn_1.0_Nb_1.5_O_7_ 170 nm thick	random	240 @1 MHz 120 @1 MHz	55% @40.8 V 28% @20.4 V	0.0022 0.0023	1617 820	[14]
PST 200 nm thick	100	25 @1 MHz 12.5 @1 MHz	60.1% @ 5 V 37.8% @2.5 V	0.0240 0.0303	29.5 18.9	[35]
Pb_0.4_Sr_0.6_Zr_0.52_Ti_0.48_O_3_ 400 nm thick	random	37.5 @1 MHz	48% @15 V	0.0128	24	[36]
BTO 300 nm thick	001	100 @5 kHz 50 @5 kHz	25.6% @30 V 15% @15 V	0.00256 0.003	12.2 7.14	[26]
PZT/BMT 640 nm thick	110	20 @1 kHz 10 @1 kHz	56.52% @13 V 38.1% @6.5 V	0.0282 0.0381	12.03 8.106	[37]
PZT/BST 300 nm thick	110	40 @1 MHz 20 @1 MHz	41.1% @12 V 20.2% @ 6 V	0.0103 0.0101	4.11 2.02	[38]
Pb_0.6_Ba_0.4_ZrO_3_ 200 nm thick	001	50 @1 MHz 25 @1 MHz	43% @10 V 26.8% @5 V	0.0086 0.0107	61.43 38.28	[39]

## Data Availability

Data will be made available on request.

## References

[1] Wu J., Zhu J., Xiao D., Zhu J., Tan J., Zhang Q. (2007). Preparation and Properties of Highly (100)-Oriented Pb (Zr_0.2_Ti_0.8_)O_3_ Thin Film Prepared by Rf Magnetron Sputtering with a PbOx Buffer Layer. J. Appl. Phys..

[2] Li R., Xu D., Du C., Ma Q., Zhang F., Liang X., Wang D., Shi Z., Liu W., Zhou D. (2024). Giant Dielectric Tunability in Ferroelectric Ceramics with Ultralow Loss by Ion Substitution Design. Nat. Commun..

[3] Wang J., Yang T., Wei K., Li G., Chen S. (2012). Bi-Tunable Dielectric Constant of Antiferroelectric PZT Ceramics Under DC Electric Field. J. Am. Ceram. Soc..

[4] Peng B., Fan H., Zhang Q. (2013). High Tunability in (111)-Oriented Relaxor Pb_0.8_Ba_0.2_ZrO_3_ Thin Film with Antiferroelectric and Ferroelectric Two-Phase Coexistence. J. Am. Ceram. Soc..

[5] James A.R., Kumar A., Prasad V.V.B., Kamat S.V., Singh V., Ghoshal P., Pandey A. (2018). Tunability, Ferroelectric and Leakage Studies on Pulsed Laser Ablated (Pb_0.92_La_0.08_)(Zr_0.60_Ti_0.40_)O_3_ Thin Films. Mater. Chem. Phys..

[6] Jakoby R., Scheele P., Muller S., Weil C. (2004). Nonlinear Dielectrics for Tunable Microwave Components. Proceedings of the 15th International Conference on Microwaves, Radar and Wireless Communications (IEEE Cat. No.04EX824).

[7] Pervez N.K., Hansen P.J., York R.A. (2004). High Tunability Barium Strontium Titanate Thin Films for Rf Circuit Applications. Appl. Phys. Lett..

[8] Aymen S., Mascot M., Jomni F., Carru J.-C. (2019). High Tunability in Lead-Free Ba_0.85_Sr_0.15_TiO_3_ thick Films for Microwave Tunable Applications. Ceram. Int..

[9] Liu S.W., Weaver J., Yuan Z., Donner W., Chen C.L., Jiang J.C., Meletis E.I., Chang W., Kirchoefer S.W., Horwitz J. (2005). Ferroelectric (Pb,Sr)TiO_3_ Epitaxial Thin Films on (001) MgO for Room Temperature High-Frequency Tunable Microwave Elements. Appl. Phys. Lett..

[10] Calzada M.L., Bretos I., Jiménez R., Ricote J., Mendiola J., García-López J., Respaldiza M.A. (2005). Chemical Solution Deposition of (Pb_1− *x*_Ca*_x_*)TiO_3_ Thin Films with *x* ∼0.5 as New Dielectrics for Tunable Components and Dynamic Random Access Memories. J. Am. Ceram. Soc..

[11] Lu S.G., Xu Z.K. (2006). Tunability and Permittivity-Temperature Characteristics of Highly (100) Oriented Compositionally Graded (Ba_0.7_Sr_0.3_)(Sn_x_Ti_1−x_)O_3_ Thin Films Grown by Pulse-Laser Deposition. Appl. Phys. Lett..

[12] Ballan H., Declercq M., Krummenacher F. (1994). Design and Optimization of High Voltage Analog and Digital Circuits Built in a Standard 5 V CMOS Technology. Proceedings of the IEEE Custom Integrated Circuits Conference—CICC ’94.

[13] Albrecht M., Erlbacher T., Bauer A.J., Frey L. (2019). Improving 5V Digital 4H-SiC CMOS ICs for Operating at 400 °C Using PMOS Channel Implantation. Mater. Sci. Forum.

[14] Lu J., Stemmer S. (2003). Low-Loss, Tunable Bismuth Zinc Niobate Films Deposited by Rf Magnetron Sputtering. Appl. Phys. Lett..

[15] Luo B., Xu Y., Zhang F., Wang T., Yao Y. (2020). Dielectric Tunability Properties in (110)-Oriented Epitaxial 0.5Ba(Ti_0.8_Zr_0.2_)O_3_-0.5(Ba_0.7_Ca_0.3_)TiO_3_ Thin Films Prepared by PLD Method. Materials.

[16] Hao L., Yang Y., Huan Y., Cheng H., Zhao Y.-Y., Wang Y., Yan J., Ren W., Ouyang J. (2021). Achieving a High Dielectric Tunability in Strain-Engineered Tetragonal K_0.5_Na_0.5_NbO_3_ Films. Npj Comput. Mater..

[17] Hu G., Gao F., Liu L., Xu B., Liu Z. (2012). Microstructure and Dielectric Properties of Highly Tunable Ba_0.6_Sr_0.4_TiO_3_/MgO/Al_2_O_3_/ZnO Composite. J. Alloys Compd..

[18] Ahmed A., Goldthorpe I.A., Khandani A.K. (2015). Electrically Tunable Materials for Microwave Applications. Appl. Phys. Rev..

[19] Lin Y., Chen X., Liu J., Yuan Z., Collins G., Chen C.L., Jiang J.C., Meletis E.I., Chen C.L.P., Bhalla A. (2008). Highly Epitaxial Ferroelectric Lead Strontium Titanate ((Pb,Sr)TiO_3_) Thin Films With Extra Large Dielectric Tunability: A Good Candidate For Room Temperature Tunable Microwave Elements. Integr. Ferroelectr..

[20] Wang F., Ma W. (2019). Phase Stability and Dielectric Properties of (011) Epitaxial (Ba_0.6_Sr_0.4_)TiO_3_ Films. J. Appl. Phys..

[21] Ambika D., Kumar V., Tomioka K., Kanno I. (2012). Deposition of PZT Thin Films With (001), (110), and (111) Crystallographic Orientations And Their Transverse Piezoelectric Characteristics. Adv. Mater. Lett..

[22] Chen L., Nagarajan V., Ramesh R., Roytburd A.L. (2003). Nonlinear Electric Field Dependence of Piezoresponse in Epitaxial Ferroelectric Lead Zirconate Titanate Thin Films. J. Appl. Phys..

[23] Mtebwa M., Tagantsev A.K., Setter N. (2014). Effect of Elastic Compliances and Higher Order Landau Coefficients on the Phase Diagram of Single Domain Epitaxial Pb(Zr,Ti)O_3_ (PZT) Thin Films. AIP Adv..

[24] Haun M.J., Furman E., Jang S.J., Cross L.E. (1989). Thermodynamic Theory of the Lead Zirconate-Titanate Solid Solution System, Part V: Theoretical Calculations. Ferroelectrics.

[25] Chang W., Gilmore C.M., Kim W.-J., Pond J.M., Kirchoefer S.W., Qadri S.B., Chirsey D.B., Horwitz J.S. (2000). Influence of Strain on Microwave Dielectric Properties of (Ba,Sr)TiO_3_ Thin Films. J. Appl. Phys..

[26] Zhang W., Cheng H., Yang Q., Hu F., Ouyang J. (2016). Crystallographic Orientation Dependent Dielectric Properties of Epitaxial BaTiO_3_ Thin Films. Ceram. Int..

[27] Noheda B., Gonzalo J.A., Cross L.E., Guo R., Park S.-E., Cox D.E., Shirane G. (2000). Tetragonal-to-Monoclinic Phase Transition in a Ferroelectric Perovskite: The Structure of PbZr_0.52_Ti_0.48_O_3_. Phys. Rev. B.

[28] Aruchamy N., Schenk T., Girod S., Glinsek S., Defay E., Granzow T. (2022). Influence of Substrate Stress on In-Plane and out-of-Plane Ferroelectric Properties of PZT Films. J. Appl. Phys..

[29] Aruchamy N., Schenk T., Kovacova V., Glinsek S., Defay E., Granzow T. (2021). Influence of Tensile vs. Compressive Stress on Fatigue of Lead Zirconate Titanate Thin Films. J. Eur. Ceram. Soc..

[30] Scott J.F., Gardner J. (2018). Ferroelectrics, Multiferroics and Artifacts: Lozenge-Shaped Hysteresis and Things That Go Bump in the Night. Mater. Today.

[31] Chan N.Y., Wang D.Y., Wang Y., Dai J.Y., Chan H.L.W. (2014). The Structural and In-Plane Dielectric/Ferroelectric Properties of the Epitaxial (Ba, Sr)(Zr, Ti)O_3_ Thin Films. J. Appl. Phys..

[32] Elissalde C., Ravez J. (2001). Ferroelectric Ceramics: Defects and Dielectric Relaxations. J. Mater. Chem..

[33] Yuan M., Zhang W., Wang X., Pan W., Wang L., Ouyang J. (2013). In Situ Preparation of High Dielectric Constant, Low-Loss Ferroelectric BaTiO_3_ Films on Si at 500 °C. Appl. Surf. Sci..

[34] Zhang W., Gao Y., Kang L., Yuan M., Yang Q., Cheng H., Pan W., Ouyang J. (2015). Space-Charge Dominated Epitaxial BaTiO_3_ Heterostructures. Acta Mater..

[35] Kim K. (2004). Dielectric Properties of Highly (100) Oriented (Pb_0.5_, Sr_0.5_)TiO_3_ Thin Films Grown on LaNiO_3_ Electrodes. Thin Solid Film..

[36] Shao Q.-Y., Li A.-D., Xia Y.-D., Wu D., Liu Z.-G., Ming N.-B. (2006). Strontium-Modified Lead Zirconate Titanate Thin Films for Electrically Tunable Device Applications. J. Appl. Phys..

[37] Wu Z., Zhou J., Chen W., Shen J., Yang H., Zhang S., Liu Y. (2016). Improvement in Temperature Dependence and Dielectric Tunability Properties of PbZr_0.52_Ti_0.48_O_3_ Thin Films Using Ba(Mg_1/3_Ta_2/3_)O_3_ Buffer Layer. Appl. Surf. Sci..

[38] Dong H., Jian J., Li H., Jin D., Chen J., Cheng J. (2017). Improved Dielectric Tunability of PZT/BST Multilayer Thin Films on Ti Substrates. J. Alloys Compd..

[39] Wu M.-H., Wu J.-M. (2005). Lead Barium Zirconate Perovskite Films for Electrically Tunable Applications. Appl. Phys. Lett..

